# Bridging the Compliance Gap: An Assessment of Dietary and Physical Activity Adherence Among Type 2 Diabetes Patients in Kericho County, Kenya

**DOI:** 10.1155/jdr/1357263

**Published:** 2025-12-29

**Authors:** Florence Wandia, Joel Wanzala

**Affiliations:** ^1^ Public Health Department, University of Kabianga, Kabianga, Kericho County, Kenya

**Keywords:** adherence, compliance, dietary recommendations, Kenya, lifestyle modification, physical activity recommendations, Type 2 diabetes

## Abstract

Adherence to lifestyle modification recommendations plays a significant role in the management of diabetes mellitus, which commonly affects elderly groups. Low or nonadherence to dietary and physical activity recommendations is a major problem that retrogresses efforts invested in diabetes care and management. This subject is underexplored in Kenya, with no existing study conducted in Kericho County. The study is aimed at bridging the existing compliance gap through assessment of dietary and physical activity adherence among Type 2 diabetes patients aged 40+ years in Kericho County, Kenya. A hospital‐based cross‐sectional study involving 207 type 2 diabetes patients at Kericho County Referral Hospital using a pretested and structured interviewer‐administered questionnaire was conducted. Validated and customized perceived dietary adherence questionnaire (PDAQ) and global physical activity questionnaire (GPAQ) were utilized. Data on sociodemographic and behavioral characteristics, and diet and exercise were collected from selected respondents through systematic random sampling technique. Collected data were analyzed using SPSS Version 26. Bivariate and multivariate logistic regression was performed to identify factors influencing adherence, with significance set at < 0.05. Results showed that 51.7% and 35.3% of the respondents were adherent to recommended dietary and physical activity, respectively. Being aged ≥ 70 years (AOR = 2.13, 95% CI: 1.01–4.87, *p* = 0.02), postprimary education (AOR = 1.71, 95% CI: 1.39–5.28, *p* = 0.02), absence of comorbidities (AOR = 1.68, 95% CI: 1.33–1.08, *p* = 0.01), and absence of complications (AOR = 1.57, 95% CI: 1.32–1.96, *p* = 0.03) had higher likelihood of adherence to dietary recommendations. Unmarried patients (AOR = 0.22, 95% CI: 0.07–0.68, *p* = 0.008) and lack of family support (AOR = 0.51, 95% CI: 0.31–0.91, *p* = 0.01) were significantly associated with lower adherence to dietary recommendations. Higher odds of physical activity adherence were associated with postprimary education (AOR = 1.75, 95% CI: 1.27–3.18, *p* = 0.03) and diabetes duration of > 10 years (AOR = 1.52, 95% CI: 1.03–2.13, *p* = 0.04), while lower among patients aged ≥ 70 years (AOR = 0.64, 95% CI: 0.29–0.87, *p* = 0.02) and lacked family support (AOR = 0.54, 95% CI: 0.37–0.85, *p* = 0.04). These findings underscore urgent need for targeted and context‐specific tailored health education, promotion of family support and involvement, and designing of individualized lifestyle modification plan that integrated functional disparities for sustainable adherence and improved diabetes outcomes.

## 1. Background Information

Diabetes mellitus (DM) is a serious metabolic disorder and public health problem globally characterized by hyperglycemia associated with inadequate insulin production and/or ineffectiveness of produced insulin [1]. According to a 2024 report by the International Diabetes Federation (IDF) Diabetes Atlas, Type 2 diabetes (T2D) accounts to approximately over 90% of all diabetes cases globally, and while high‐income countries report relatively higher prevalence rates, low‐ and middle‐income countries account for more than 81% of the global diabetes cases attributed to their larger population sizes [2]. Over decades, studies have reported a significant rise in the T2D trend. It was estimated that approximately 382 million people had DM in 2013, 425 million in 2017, and about 589 million in 2024; by 2050, it is projected to rise to 853 million (this represents a 45% increase) [3, 4]. In 2024, DM was reported to be a major cause of death among diabetic patients, with over 3.4 million deaths [5]. In Africa, the IDF Diabetes Atlas 2024 reports projecting a 145% rise by 2050 from 24.6 million in 2024. It is estimated that DM accounted for approximately $1.015 trillion in global health expenditure in 2024, which represented approximately 338% increase over the past 17 years [2].

Lifestyle modifications such as healthy dietary habits and physical activity play a critical role in achieving optimal glycemic control and overall T2D management and prevention of diabetes‐related complications. Studies have reported that healthy dietary practices and physical activity could result in approximately 5.7% weight loss to successfully maintain optimal blood sugar control while subsequently reducing insulin demand and increasing tissue sensitivity to insulin [6, 7]. Therefore, adherence to dietary and physical activity recommendations is crucial among T2D patients for optimal diabetes management. Adherence to lifestyle modifications (diet and exercise) results in significant improvement in glycemic levels, which lessens risks to the disease′s micro‐ and macrovascular complications such as blood pressure and lipid abnormalities while reducing diabetes‐related mortality [8, 9].

Nonadherence to prescribed dietary and physical activity recommendations is a major barrier to optimal management and prevention of T2D. Studies have reported suboptimal adherence to lifestyle modifications as a major public health issue, especially among LMICs, including Kenya, affecting efforts and health interventions put in place to address and prevent T2D and related diabetic complications [10, 11]. In Kenya, there is limited data and studies on adherence to dietary and physical activity among T2D patients aged 40+ years, a higher T2D risk age group. Few existing studies have reported lower adherence levels to recommended lifestyle modifications, with approximately 22.4%–36% and 18.9%–31.7% adhering to dietary and physical activity guidelines, respectively [12, 13]. Several studies have reported that adherence to lifestyle modification recommendations is significantly influenced by sociodemographic, rapid socioeconomic development, and urbanization, among others [14, 15]. While adherence to lifestyle modifications among diabetic patients has been widely documented globally and in other African settings, there is limited empirical data, particularly in Kericho County, where there is no published report or study on adherence to lifestyle modification despite variations in sociocultural practices, dietary patterns, and a devolved health system. This presents a significant knowledge gap that warrants further exploration. Consequently, this study was aimed at bridging this existing knowledge through investigating adherence to diet and physical activity among diabetic patients in Kericho County, Kenya.

## 2. Materials and Methods

### 2.1. Study Design and Population

A facility‐based cross‐sectional study was conducted from December 2024 to February 2025, involving 207 T2D patients attending Kericho County Referral Hospital (KCRH) in Kericho County, Kenya. The KCRH is located in Kericho town, and it is the main healthcare facility in Kericho County with a catchment population across the county, including specialized diabetic care and management services. The study population was T2D patients who attended the diabetic clinic at KCRH during the study period. Individuals aged ≥ 40 years, previously diagnosed with T2D for at least 3 months, and provided written informed consent were included. Previous studies have reported the highest risk, complication rates, and lifestyle‐related metabolic changes among T2D patients aged ≥ 40 years [6, 7]. Additionally, patients with mental impairment, diagnosed with acute illness, gestational diabetes, or pregnancy, and requiring intense specialized care or monitoring were excluded from the study.

### 2.2. Sample Size Determination and Sampling Technique

Sample size was determined using Andrew Fisher′s formula, considering *n* = *Z*2*p* *q*/*d*2; where *n* was the required sample size, *p* was the expected prevalence of adherence to diet and physical activity, assumed to be 50% since there was no similar study in this area; that is, *q* = 1 − *p*; and *d* was margin error or precision, which resulted in 384 patients. However, since the target population was less than 10,000 (the estimate was 420 T2D patients), we adjusted the sample using the correction formula (nf = No/1 + No/*N*), and 10% attrition was added, resulting in a final sample of 220 patients. Out of the calculated sample size of 220, the study successfully recruited 207 participants, representing 94.1% of the target. A systematic random sampling technique was adopted to select the T2D patients by randomly selecting the first participant and then including every second patient thereafter based on daily clinic attendance order.

### 2.3. Data Collection Tool and Measurements

A structured questionnaire adopted from previous similar studies, customized based on patient follow‐up data and validated tools, was used to collect data from the respondents through face‐to‐face interviews by trained research assistants. The questionnaire comprised three sections: (i) sociodemographic characteristics, (ii) dietary questions, and (iii) physical activity questions. Sociodemographic characteristics included age, gender, religion, residence, marital status, education, employment status, T2D duration, alcohol consumption, smoking status, comorbidities (such as hypertension, cardiovascular disease, kidney disease, and obesity), and diabetes‐related complications (such as neuropathy, nephropathy, retinopathy, and diabetic foot ulcers). For dietary adherence assessment, questions were adopted and contextually customized from the validated 9‐point perceived dietary adherence questionnaire (PDAQ) to fit study setting. PDAQ questions were based on 7‐point Likert responses, which focused on 7‐day assessments, phrased as “on how many of the last 7 days did you…?” as adopted by previous similar studies [16, 17]. The tool inherently assumes participants′ behaviors over the preceding 7 days were typical or habitual. High scores in the scale (at least 4 days per week) were considered as adherence to dietary, with exception for Items 4 and 9, which were reversed questions (where high score represented nonadherence). The last section assessed physical activity, contextually modified based on validated global physical activity questionnaire (GPAQ) tool, WHO physical activity recommendations [18], and previous similar studies [19, 20]. The section included questions on whether they engage in exercise (yes/no), type of exercise, duration, and frequency, scored to either adherence or nonadherence to physical activity guidelines. Additionally, all tools adopted in this study were contextually modified and were also publicly available for academic and noncommercial research purposes.

### 2.4. Data Quality Assurance

The data collection tool was robustly designed and pretested in Kapkatet Subcounty Hospital (KSCH) using 22 T2D patients, resulting in a Cronbach′s alpha coefficient of 0.728. Key and necessary adjustments were made to the questionnaire prior to the main data collection. Research assistants (data collectors) were trained for 4 days on the data collection tool, Open Data Kit (ODK) operation, respondents′ engagement, and interviewing approaches. Actual data collection was conducted under the vigorous supervision of experienced principal investigators. The collected and ODK‐downloaded data were carefully checked for completeness, accuracy, relevancy, and clarity by the principal investigators.

### 2.5. Statistical Analysis

Collected, downloaded, and complete data were cleaned, coded, and statistically analyzed using IBM Statistical Package for Social Sciences (SPSS) Version 26. Descriptive statistics such as frequency, percentages, mean, and standard deviation (SD) were used to summarize sociodemographic characteristics and lifestyle modifications (dietary and physical activity). PDAQ responses were scored and transformed, with scores ≥ 36 indicated adherence while < 36 represented nonadherence to dietary recommendation [21]. Engagement to physical activity, exercise duration, and frequency per week were scored, and a total score of ≥ 5 indicated adherence while less than 5 for nonadherence. Marital status was merged into unmarried (single, widowed, and divorced/separated) versus married to reduce sparse categories and improve statistical power for interpretable regression models estimates, while religion was excluded due to small number of subcategory (Muslim) participants produced underpowered and noninformative estimates. Binomial logistic regression model was used to assess estimate crude (unadjusted) odds ratios, while multivariate logistic regression model assessed adjusted odds ratios with corresponding 95% confidence interval (CI). This approach was adopted to control potential confounders while avoiding bias due to selective variable exclusion. *p* value of 0.05 was considered to be statistically significant.

### 2.6. Ethical Considerations

The study was approved by the Institutional and Scientific Review Board of University of Eastern Africa, Baraton (UEAB/ISERC/01/09/2024) and obtained study permit from the National Commission for Science, Technology, and Innovation (NACOSTI/P/24/41338). Study permission was obtained from KCRH administration. The participants were briefed about the study′s objectives and areas of assessment, and written consent was taken from the participants before data collection. The right of the participants who do not want to participate in the study was respected. Confidentiality and privacy of the respondents were ensured, as no identifiers such as names, address, and other personal details were recorded during data collection.

## 3. Results

### 3.1. Sociodemographic and Behavioral Characteristics of the Study Participants

Table 1 shows that the majority of the respondents aged 40–54 years (44.4%), were males (51.2%), were Christians (95.2%), and resided in rural settings (56.5%). More than three‐quarters (79.2%) attained postprimary education while nine in 10 (90.3%) respondents were married. The majority of the participants were employed (77.3%), had lived with T2D for 10 years or less (66.7%), never consumed alcohol (71.5%), and were nonsmokers (77.3%). More than two‐fifths of the respondents reported to have comorbidity (48.8%) and diabetes‐related complications (43.5%). Additionally, the majority (71.0%) reported that they had family support.

**Table 1:** Sociodemographic and behavioral characteristics of the study participants.

**Sociodemographic variables**	**Frequency (** **n** = 207**)**	**Percentage (%)**
Age (years)		
40–54	92	44.4
55–69	83	40.1
≥ 70	32	15.5
*M* *e* *a* *n* ± *S* *D*	57.7 ± 11.2	
Gender		
Male	106	51.2
Female	101	48.8
Religion		
Christians	197	95.2
Muslims	10	4.8
Residence		
Rural	117	56.5
Urban	90	43.5
Educational attainment		
Basic education	43	20.8
Postprimary education	164	79.2
Marital status		
Married	187	90.3
Unmarried	20	9.7
Employment status		
Employed	160	77.3
Unemployed	47	22.7
T2D duration		
≤ 10 years	138	66.7
> 10 years	69	33.3
Alcohol consumption		
Yes	56	28.5
No	148	71.5
Smoking status		
Yes	47	22.7
No	160	77.3
Presence of comorbidity		
Yes	101	48.8
No	106	51.2
Presence of diabetes‐related complication(s)		
Yes	90	43.5
No	117	56.5
Family support		
Yes	147	71.0
No	60	29.0

### 3.2. Prevalence of Adherence to Dietary Recommendations

The PDAQ showed that more than half (51.7%, *n* = 107) of the T2D patients adhered to dietary guidelines, while 48.3% (*n* = 100) were nonadherent. PDAQ mean scores across the 7‐day recall period were relatively following a healthful eating plan such as in the diabetic plate (M = 4.52, SD = 1.05), consuming carbohydrate‐containing foods with a low glycemic index/whole grain products/unprocessed food (M = 4.10, SD = 0.83), and eating fruit and vegetable based on food guide/diabetic plate (M = 4.08, SD = 0.76). However, high mean scores of high‐fat foods (M = 4.12, SD = 1.01) and high‐sugar consumption (M = 3.96, SD = 1.07) were reported, although they were both reversely scored (Table 2).

**Table 2:** Perceived dietary adherence questionnaire (PDAQ) scores for Type 2 diabetes patients.

**Item**	**Mean, SD**
On how many of the last 7 days have you followed a healthful eating plan such as in the diabetic plate?	4.52 (1.05)
On how many of the last 7 days did you eat the number of fruit and vegetable servings you are supposed to eat based on food guide/diabetic plate?	4.08 (0.76)
On how many of the last 7 days did you eat carbohydrate‐containing foods with a low glycemic index/whole grain products/unprocessed food? (Example: Ugali, chapati, potatoes, dried beans, lentils, low‐fat dairy products)	4.10 (0.83)
On how many of the last 7 days did you eat foods high in sugar, such as cakes, sweets, biscuits, cookies, desserts, and candies? ^∗^	3.96 (1.07)
On how many of the last 7 days did you eat foods high in fiber, such as oatmeal, high‐fiber cereals, and whole‐grain breads? (Example: Weetabix Whole Grain, brown bread, etc.)	4.01 (0.93)
On how many of the last 7 days did you space carbohydrates evenly throughout the day?	4.05 (0.99)
On how many of the last 7 days did you eat fish or other foods high in omega‐3 fats, for example, soybeans?	4.08 (0.95)
On how many of the last 7 days did you eat foods that contained or was prepared with healthy oils, such as olive oil, coconut/palm oil, and sunflower oil?	4.04 (1.03)
On how many of the last 7 days did you eat foods high in fat (such as high‐fat dairy products, fatty meat, fried foods, or deep‐fried foods)? ^∗^	4.12 (1.01)
Overall adherence (*n*, %)	
Nonadherent	100 (48.3)
Adherent	107 (51.7)

*Note:* Asterisk (∗) means reversely scored, score < 36 = nonadherent, score ≥ 36 = adherent.

Abbreviation: SD, standard deviation.

### 3.3. Adherence to Physical Activity Recommendations

Table 3 reveals that overall adherence to physical activity recommendations among T2D patients was 35.3% (*n* = 73), while 64.7% (*n* = 134) were nonadherent. The majority of the respondents engaged in exercise (91.3%). Out of those who engaged in exercise, 51.3% engaged in daily exercise for at least 30 min, and three‐fifths (60.8%) engaged in exercise less than five times a week. Additionally, majority of the T2D patients engaged in cycling (74.1%), climbing stairs (38.6%), and running (38.1%).

**Table 3:** Adherence to physical activity recommendations among Type 2 diabetes patients.

**PA variable**	**Frequency (** **n** **)**	**Percentage (%)**
Exercise engagement		
Yes	189	91.3
No	18	8.7
Daily exercise duration (*n* = 189)		
< 30 min	92	48.7
≥ 30 min	97	51.3
Weekly exercise frequency (*n* = 189)		
< 5 days	115	60.8
≥ 5 days	74	39.2
Type of exercise		
Brisk walking	140	74.1
Cycling	46	24.3
Running	72	38.1
Climbing stairs	73	38.6
Swimming	54	28.6
Other	4	2.1
Overall adherence		
Nonadherent	134	64.7
Adherent	73	35.3

*Note:* Other = digging (land tilling) and rope skipping.

### 3.4. Factors Influencing Adherence to Recommended Lifestyle Modifications

Table 4 shows bivariate and multinomial logistic regression results on association between adherence to dietary recommendation and sociodemographic and behavioral factors. The results revealed that individuals aged ≥ 70 years were significantly three times more likely to adhere to dietary recommendation compared with those aged 40–54 years (AOR = 2.13, 95% CI: 1.01–4.87, *p* = 0.02). Similarly, patients who had attained postprimary education (AOR = 1.71, 95% CI: 1.39–5.28, *p* = 0.02), absence of comorbidities (AOR = 1.68, 95% CI: 1.33–2.08, *p* = 0.01), and absence of complications (AOR = 1.57, 95% CI: 1.32–1.96, *p* = 0.03) had higher likelihood of adherence to dietary recommendations. Additionally, unmarried patients (AOR = 0.22, 95% CI: 0.07–0.68, *p* = 0.008) and lack of family support (AOR = 0.51, 95% CI: 0.31–0.91, *p* = 0.01) were significantly associated with lower adherence to dietary recommendations. Multinomial logistic regression results revealed that patients aged ≥ 70 years (AOR = 0.64, 95% CI: 0.29–0.87, *p* = 0.02) and lacked family support (AOR = 0.54, 95% CI: 0.37–0.85, *p* = 0.04) were significantly less likely to adhere to recommended physical activity. Higher educational attainment (postprimary) (AOR = 1.75, 95% CI: 1.27–3.18, *p* = 0.03) and diabetes duration of > 10 years (AOR = 1.52, 95% CI: 1.03–2.13, *p* = 0.04) were significantly associated with higher odds of adherence to recommended physical activity. Similarly, patients without comorbidities (AOR = 1.49, 95% CI: 1.26–2.90, *p* = 0.02) or diabetes‐related complications (AOR = 1.62, 95% CI: 1.17–3.02, *p* = 0.013) were more likely to adhere to recommended physical activity (Table 5).

**Table 4:** Sociodemographic and behavioral factors influencing adherence to dietary recommendations among Type 2 diabetes patients.

	**Nonadherent**	**Adherent**	**COR**	**95% CI**	**p** **value**	**AOR**	**95% CI**	**p** **value**
	**n** **(%)**	**n** **(%)**						
Age (years)								
40–54	48 (23.2%)	44 (21.3%)	ref	—	—	—	—	—
55–69	42 (20.3%)	41 (19.8%)	1.07	0.59–1.93	0.84	0.44	0.19–1.05	0.07
≥ 70	10 (4.8%%)	22 (10.6%)	2.40	1.02–5.63	0.04 ^∗^	2.13	1.01–4.87	0.02 ^∗^
Gender								
Male	56 (27.1%)	50 (24.2%)	ref	—	—	—	—	—
Female	44 (21.3%)	57 (27.5%)	1.42	0.80–2.53	0.23	1.39	0.78–2.49	0.27
Residence								
Rural	59 (28.5%)	58 (28.0%)	ref	—	—	—	—	—
Urban	41 (19.8%)	49 (23.7%)	1.37	0.74–2.52	0.31	1.39	0.75–2.57	0.30
Educational attainment								
Basic education	17 (8.2%)	26 (12.6%)	ref	—	—	—	—	—
Post‐primary education	83 (40.1%)	81 (39.1%)	1.64	1.31–4.33	0.003	1.71	1.39–5.28	0.02 ^∗^
Marital status								
Married	85 (41.1%)	102 (49.3%)	ref	—	—	—	—	—
Unmarried	15 (7.5%)	5 (2.4%)	0.23	0.08–0.70	0.01 ^∗^	0.22	0.07–0.68	0.008 ^∗^
Employment status								
Employed	79 (38.2%)	81 (39.1%)	ref	—	—	—	—	—
Unemployed	21 (10.1%)	26 (12.6%)	1.14	0.54–2.39	0.72	1.12	0.53–2.36	0.78
DM duration								
≤ 10 years	70 (33.8%)	68 (32.9%)	ref	—	—	—	—	—
> 10 years	30 (14.5%)	39 (18.8%)	1.08	0.57–2.02	0.81	1.11	0.59–2.09	0.75
Alcohol consumption								
Yes	30 (14.5%)	29 (14.0%)	ref	—	—	—	—	—
No	70 (33.8%)	78 (37.7%)	1.39	0.72–2.69	0.32	1.37	0.71–2.65	0.35
Smoking status								
Yes	20 (9.7%)	27 (13.0%)	ref	—	—	—	—	—
No	80 (38.6%)	80 (38.6%)	0.84	0.41–1.76	0.65	0.77	0.36–1.62	0.49
Presence of comorbidity								
Yes	42 (20.3%)	59 (28.5%)	ref	—	—	—	—	—
No	58 (28.0%)	48 (23.2%)	1.64	1.36–1.13	< 0.001	1.68	1.33–2.08	0.01 ^∗^
Presence of complication(s)								
Yes	36 (17.4%)	54 (26.1%)	ref	—	—	—	—	—
No	64 (30.9%)	53 (25.6%)	1.56	1.31–1.99	0.04	1.57	1.32–1.96	0.03 ^∗^
Family support								
Yes	76 (36.7%)	71 (34.3%)	ref	—	—	—	—	—
No	24 (11.6%)	36 (17.4%)	0.69	0.48–0.95	0.02	0.51	0.31–0.91	0.01 ^∗^

Abbreviations: AOR, adjusted odds ratio; CI, confidence interval; COR, crude odds ratio; *n*, frequency; ref, reference category.

^∗^Statistically significant (< 0.05).

**Table 5:** Sociodemographic and behavioral factors influencing adherence to recommended physical activity.

	**Nonadherent**	**Adherent**	**COR**	**95% CI**	**p** **value**	**AOR**	**95% CI**	**p** **value**
	**n** **(%)**	**n** **(%)**						
Age (years)								
40–54	65 (31.4%)	27 (13.0%)	ref	—	—	—	—	—
55–69	54 (26.1%)	29 (14.0%)	0.67	0.39–0.93	0.65	0.54	0.34–0.84	0.72
≥ 70	15 (7.2%)	17 (8.2%)	0.73	0.34–0.91	0.04 ^∗^	0.64	0.29–0.87	0.02 ^∗^
Gender								
Male	64 (30.9%)	42 (20.3%)	ref	—	—	—	—	—
Female	70 (33.8%)	31 (15.0%)	0.65	0.36–1.17	0.15	0.66	0.36–1.21	0.18
Residence								
Rural	73 (35.3%)	44 (21.3%)	ref	—	—	—	—	—
Urban	61 (29.5%)	29 (14.0%)	0.90	0.48–1.69	0.74	0.92	0.49–1.74	0.81
Educational attainment								
Basic education	24 (11.6%)	19 (9.2%)	ref	—	—	—	—	—
Postprimary education	110 (53.1%)	54 (26.1%)	1.60	1.29–1.94	0.01	1.75	1.27–3.18	0.03 ^∗^
Marital status								
Married	122 (58.9%)	65 (31.4%)	ref	—	—	—	—	—
Unmarried	12 (5.8%)	8 (3.9%)	1.00	0.37–2.71	0.99	0.87	0.31–2.41	0.78
Employment status								
Employed	105 (50.7%)	55 (26.6%)	ref	—	—	—	—	—
Unemployed	29 (14.0%)	18 (8.7%)	0.85	0.40–1.79	0.67	0.85	0.40–1.81	0.68
DM duration								
≤ 10 years	92 (44.4%)	46 (22.2%)	ref	—	—	—	—	—
> 10 years	42 (20.3%)	27 (13.0%)	1.21	1.01–2.11	0.03	1.52	1.03–2.13	0.04 ^∗^
Alcohol consumption								
Yes	34 (16.4%)	25 (12.1%)	ref	—	—	—	—	—
No	100 (48.3%)	48 (23.2%)	0.75	0.37–1.46	0.39	0.77	0.39–1.51	0.44
Smoking status								
Yes	27 (13.0%)	20 (9.7%)	ref	—	—	—	—	—
No	107 (51.7%)	53 (25.6%)	0.69	0.33–1.46	0.34	0.71	0.33–1.53	0.38
Presence of comorbidity								
Yes	58 (28.0%)	43 (20.8%)	ref	—	—	—	—	—
No	76 (36.7%)	30 (14.5%)	1.51	1.28–1.93	0.02 ^∗^	1.49	1.26–2.90	0.02 ^∗^
Presence of complication(s)								
Yes	62 (30.0%)	28 (13.5%)	ref	—	—	—	—	—
No	72 (34.8%)	45 (21.7%)	1.68	1.21–3.11	0.019 ^∗^	1.62	1.17–3.02	0.013 ^∗^
Family support								
Yes	74 (45.4%)	53 (25.6%)	ref	—	—	—	—	—
No	40 (19.3%)	20 (9.7%)	0.71	0.28–0.93	0.01	0.54	0.37–0.85	0.04 ^∗^

Abbreviations: AOR, adjusted odds ratio; CI, confidence interval; COR, crude odds ratio; *n*, frequency; ref, reference category.

^∗^Statistically significant (< 0.05).

## 4. Discussion

This study revealed that more than half (51.7%) of the T2D patients adhered to dietary guidelines. This finding was consistent with a similar study conducted in Ethiopia, which found 53.2% were dietary adherent [22]. However, the current study′s finding was higher than studies in Calgary, Saudi Arabia, Hungary, and Egypt, which reported dietary adherence rates of 45%, 32.1%, 21.7%, and 5.7%, respectively [21, 23–25]. The current finding was lower than studies in Mexico (74.2%) and Iran (63.0%) [12, 26]. Similarly, a study in Nepal revealed that three‐fifths (60%) of the T2D patients were adherent to dietary recommendations [27]. This variation in findings could be explained by differences in dietary guideline dissemination strategies, patient education, healthcare access, and cultural norms. The current study found a physical activity adherence rate of 35.3% among T2D patients. Similarly, existing studies have reported lower rates of adherence to physical activity among T2D patients. A study by Du et al. reported that only a third of the patients adhered to recommended physical activity [28]. Low adherence to physical activity is often attributed to poor self‐efficacy, lack of social support, comorbidities, and limited knowledge about the benefits of exercise in diabetes control. Therefore, there is an urgent need to improve adherence to dietary and physical activity recommendations among T2D patients in Kericho County.

Although religion was excluded from regression models due to small number of Muslim participants, which produced underpowered and noninformative estimates, the multinomial logistic regression results in association between adherence to dietary recommendation and sociodemographic and behavioral factors. The results revealed that individuals aged ≥ 70 years were significantly three times more likely to adhere to dietary recommendation compared with those aged 40–69 years. This result is in agreement with the study in Spain [29]. This could be due to higher health consciousness among older adults, who are often more motivated to comply with medical and dietary guidelines as a result of greater vulnerability perception to diabetes‐related complications. Additionally, younger individuals often have more occupational responsibilities and hence busy and unstructured routines, which may result in low dietary adherence. Similar to studies by Carrasco‐Marin et al. [30] and Rodríguez‐Pérez et al. [31], the current study found that higher educational attainment (postprimary) had a higher likelihood to adhere to dietary recommendations. This could be attributed to increased awareness and knowledge among patients with higher education on the benefits and role of dietary adherence in diabetes management and prevention of diabetes‐related complications. Similarly, highly educated people can easily understand, agree with, and come across a considerable amount of information regarding recommended dietary guidelines. This study found that the absence of comorbidities or complications was significantly associated with higher dietary adherence. These findings concur with the study conducted in Nepal [27] and Ethiopia [32]. According to Feleke et al., comorbidities and complications hinder dietary adherence due to complex dietary recommendations and restrictions [33]. Individuals who were unmarried and lacked family support were less likely to adhere to dietary recommendations. These results are supported by studies in Ethiopia [32] and Pakistan [34]. Unmarried people lack the social or family support to adhere or be consistent to recommended health intervention [12]. Additionally, a possible explanation could be lack of family support might result in inadequate motivation and practical support that facilitate relapse and nonadherence. Social support, particularly from family, plays a vital role in improvement of dietary adherence due to emotional well‐being and practical support that act as motivational drive toward health intervention adherence.

The study revealed that patients aged ≥ 70 years were significantly less likely to adhere to recommended physical activity. This finding was consistent with the study in Ethiopia and Nigeria. Similarly, a study in the United States reported attributing this trend to diminished self‐efficacy and poor self‐regulation strategies among the elderly, who often cited fatigue, fear of injury, age‐related limitations, physical discomfort, and complex physical exercise recommendations as key barriers [35]. Higher educational attainment (postprimary) and diabetes duration of > 10 years were significantly associated with higher odds of adherence to recommended physical activity. These findings corroborate similar studies [7, 12]. The possible explanation could be increased knowledge of the disease and the significance of physical activity in diabetes management. Consistent with several studies [14, 27], this study found that patients without comorbidities or complications were more likely to adhere to recommended physical activity. Absence of comorbidities significantly improves mobility, physical capacity, and self‐confidence for consistent participation in physical activity. A study by Anderson and Durstine reported that additional health burdens such as hypertension or cardiovascular diseases often face chronic pain and fatigue, which limit physical activity [36]. Additionally, patients with comorbidities and complications often experience psychological burdens such as exacerbation of the disease′s symptoms, which deter physical activity engagement. Therefore, there is an urgent need for tailored physical activity interventions that fully integrate the functional limitations of T2D patients with multiple morbidities.

## 5. Strengths and Limitations of the Study

This study investigated dietary and physical adherence among T2D patients aged 40+ years, a subject that was not explored in Kericho County; hence, there was a paucity of data. The study bridged this critical gap in diabetes management efforts through insightful findings to improve adherence to dietary and physical activity recommendations. It revealed various factors that influence adherence to lifestyle modifications (diet and physical activity) in Kericho County, Kenya, providing valuable insights to address and improve adherence among patients. The study was conducted in a major healthcare facility in the county with catchment population of the entire region, hence more relevant for this study. However, this study was not free of limitations. The study used a structured questionnaire that limited participants′ responses on the subject. The study was conducted in one primary healthcare facility in the county, in patients aged 40 years or above, and only among diabetes patients who attended the facility during the time‐bound study period, which may affect generalizability of findings. Additionally, dietary and physical activity assessments were obtained by self‐reporting, and this might have introduced recall and social desirability biases to the study.

## 6. Conclusion

Adherence to lifestyle modifications (diet and physical activity) is still relatively low among T2D patients in Kericho county, Kenya. Adherence to dietary recommendations was relatively higher compared with physical activity adherence. All stakeholders including policymakers, health educationists and promoters, dieticians, and other healthcare workers should be aware of low adherence to diet and physical activity recommendations in Kericho County. Several sociodemographic and behavioral factors significantly influence adherence to recommended diet and physical activity. The study underscores urgent need for all stakeholders engaged in diabetes management to promote patient education and health promotion through provision of relevant information, to address paucity of information. Healthcare workers and other relevant stakeholders should design and tailor dietary and physical activity interventions or recommendation that consider functional limitations, while providing or promoting social support for vulnerable groups. Additionally, there is need to investigate in‐depth knowledge level of diet and exercise guidelines among the patients while assessing healthcare workers′ perspectives on the subject.

## Disclosure

Both authors have read and approved this manuscript.

## Conflicts of Interest

The authors declare no conflicts of interest.

## Author Contributions

F.W. has conceived the study, collected the data, and reviewed the manuscript. J.W. has conceived the study, analyzed the data, drafted the manuscript, and reviewed the manuscript.

## Funding

No funding was received for this manuscript.

## Data Availability

The data that support the findings of this study are available from the corresponding author upon reasonable request.
